# Dietary supplementation with herbal powder of *Elsholtzia cypriani* improves immune function in Muscovy ducks by activating endogenous antioxidant system and modulating gut microbiota

**DOI:** 10.1016/j.psj.2025.106080

**Published:** 2025-11-08

**Authors:** HuiWei Zhou, XinYe Tian, YongPeng Ma, AZheng Liang, XiaoDong Zhuang, ZhiZhi Du

**Affiliations:** a Yunnan Key Laboratory for Wild Plant Resources, Department of Economic Plants and Biotechnology, Kunming Institute of Botany, Chinese Academy of Sciences, Kunming 650201, China; b Zhaotong Academy of Agricultural Sciences, Zhaotong, Yunnan 657000, China; c Zhangzhou Changlong Agriculture and Animal Husbandry Co., Ltd, Fujian 363100, China

**Keywords:** *Elsholtzia cypriani*, Production performance, Gut microbiot, Immunity, Antioxidant activity

## Abstract

*Elsholtzia cypriani*, a traditional medicinal and edible aromatic plant, exhibits antibacterial, antioxidant, and immunomodulatory properties, making it a promising plant-based feed additive and a potential alternative to antibiotics. However, there is a lack of experimental evidence from case studies. This study investigated the effects of dietary supplementation with *E. cypriani* on Muscovy ducks. GC-MS analysis identified geranial (45.09 %) and neral (36.29 %) as the principal constituents of *E. cypriani* essential oil. In vitro bioactivity tests demonstrated significant inhibitory effects against *Escherichia coli* and *Salmonella anatum*. A feeding trial was conducted using medium-sized white-feathered Muscovy ducks, with 4 % *E. cypriani* herb powder added to their diet. The results showed that although growth performance and slaughter rate were not significantly improved, the measure effectively stimulated appetite, leading to increased feed intake and weight gain. It also significantly elevated the thymus index, suggesting it can promote immune organ development. Compared to the control group, dietary supplementation with *E. cypriani* boosted the systemic antioxidant capacity in ducks, characterized by increased glutathione peroxidase (GSH-Px) activity (*p* < 0.01) and significantly reduced malondialdehyde (MDA) content (*p* < 0.001). Furthermore, it considerably improved the inflammatory status, marked by significant reductions in pro-inflammatory cytokines IL-1β, TNF-α, and IL-6 (*p* < 0.05). Concurrently, in the cecum, supplementation resulted in significantly elevated acetic acid levels and an enrichment of fiber-degrading microbiota such as UBA11471 and *Bacteroides H massiliensis*. This suggests *E. cypriani* may promote short-chain fatty acid production by regulating the gut microbiota composition. In summary, *E. cypriani* enhances the antioxidant and anti-inflammatory capacity of Muscovy ducks by activating the endogenous antioxidant system and modulating gut microbiota, thereby improving overall immune status and production performance. This work underscores the potential of *E. cypriani* as a natural feed additive and offers insights for developing sustainable alternatives to antibiotics in poultry farming.

## Introduction

Antibiotics have been widely used as growth promoters in food animals for a long time. They are added at low doses to feed to enhance growth performance and prevent disease in livestock (Miles et al., 2006). However, this practice means unabsorbed antibiotics enter the environment through feces. This results in the accumulation of drugs in soil and water bodies (Barton, 2014). It also promotes the generation and spread of drug-resistant bacteria and genes (Glynn et al., 1998; G. Liu, et al., 2022), potentially threatening human health through the food chain (Chen et al., 2019). As these negative impacts become more evident, many countries have gradually restricted the use of antibiotics in animal feed (Castanon, 2007).

The "antibiotic restriction" policy has had a significant impact on the livestock industry. It has reduced production efficiency and increased farming costs (Brown et al., 2017). As a result, developing green, efficient, and sustainable alternatives to antibiotics has become a research priority. Current primary strategies involve systematically evaluating the efficacy of other options using metrics such as growth performance, feed conversion ratio, and gut health (Brown et al., 2017). Widely validated alternatives include enzyme preparations, organic acids, prebiotics, probiotics, herbal extracts, trace elements, and vaccines (Cheng et al., 2014; Wang et al., 2024).

Among numerous alternatives, plant-derived feed additives have attracted much attention due to their natural origins, multifunctional properties, low toxicity, and low residue (Abd El-Hack et al., 2022; Li et al., 2022; Wang et al., 2024). Traditional Chinese medicinal herbs and their extracts are not only abundant in resources, but also have the advantages of not easily inducing drug resistance and low residue risk (Cheng et al., 2014; Dhama et al., 2015; Liu et al., 2024). Currently, widely applied plant bioactive components include polyphenols, essential oils, polysaccharides, organic acids, and alkaloids (Abd El-Hack et al., 2022; Brenes and Roura, 2010; Oni and Oke, 2025). Plant essential oils, as secondary metabolites of aromatic plants, have excellent anti-inflammatory, antioxidant, and antibacterial activities, showing broad application potential in promoting animal health and growth (Liu et al., 2022; Wang et al., 2024). In addition to essential oils, the direct incorporation of aromatic plant powders has also achieved favorable outcomes. For example, rosemary leaf powder enhances antioxidant function and egg quality in laying hens (Zhang et al., 2024), while adding garlic stem powder and oregano powder boosts immune capacity and muscle protein content in fish (Xu et al., 2010).

Aromatic plants and their essential oils have garnered widespread attention due to their safety profile and minimal side effects (Li et al., 2022; Moharreri et al., 2021). Aromatic plants rich in citral, such as *Litsea cubeba* and *Cymbopogon citratus* oils, have outstanding antibacterial, anti-inflammatory, antioxidant, and insecticidal properties (Abdul et al., 2015; Bachiega and Sforcin, 2011; Hang et al., 2020; Lv et al., 2023; Wang et al., 2024). They show strong potential for development as feed additives. Studies indicate that *L. cubeba* essential oil, with a distinctive lemon aroma, enhances animal feed intake. When added to finishing pigs' diets, it also improves growth performance, blood parameters, and antioxidant capacity (Chen et al., 2023). Supplementing with new natural plant-based feed additive lemongrass essential oil (LGEO) in poultry production has improved performance, blood lipid levels, immunity, and antioxidant markers in growing quails. It also reduces intestinal pathogens, thereby enhancing the overall health status of growing quails (Boukhatem et al., 2014). Citral, when appropriately protected through encapsulation, can be used to control necrotic enteritis in chickens without significantly affecting the intestinal load of Lactobacillus (Yang et al., 2016). Furthermore, at a concentration of 40 μg/mL, citral inhibits at least 90 % of fungal growth and aflatoxin B1 production in broth and poultry feed, offering a potential alternative method for controlling aflatoxin contamination in food and feed (Han et al., 2022). Lemongrass essential oil exhibits antibacterial activity against *E. coli, Staphylococcus aureus*, and *Salmonella*, with efficacy increasing with concentration (Ebani et al., 2019). Citral also demonstrates anti-inflammatory activity by inhibiting NO production via suppression of iNOS and NF-κB (Lee et al., 2008), along with antioxidant, insecticidal, and antifungal properties that help repel insects, inhibit mold, and extend food shelf life (Li et al., 2025).

The overuse of antibiotics has led to issues such as antibiotic resistance and frequent outbreaks of foodborne illnesses. As a result, developing safe and effective feed additive resources has become an urgent need for sustainable aquaculture. *Elsholtzia cypriani,* as a traditional medicinal and edible plant, has long been used as a culinary spice in Yunnan, Guizhou, and other regions of China. It is rich in citral, with other major constituents including volatile components such as linalool and β-caryophyllene, as well as non-volatile components like flavonoids, phenols, and terpenoids. Luteolin, luteolin-7-O-*β*-ᴅ-glucopyranoside, quercetin, quercetin-3-O-*β*-ᴅ-galactopyranoside, and quercetin-3-O-*β*-ᴅ-galactoside, which were isolated and identified from *E. cypriani,* showed vigorous antioxidant activity with free radical scavenging rates exceeding 90 % at specific concentrations (Ping and Qing, 2016). The essential oil extracted from *E. cypriani* flowers significantly inhibits *Staphylococcus aureu*s and also exhibits some inhibitory effects on *Salmonella typhi* and *Klebsiella pneumoniae* (Ping et al., 2017). The ethanol total extract of *E. cypriani* exhibited certain capabilities in scavenging both DPPH and ABTS free radicals. The scavenging rates of various crude extract fractions of *E. cypriani* against DPPH and ABTS free radicals demonstrated a concentration-dependent relationship (Jian et al., 2014). *E. cypriani* shows promising antibacterial, anti-inflammatory, and antioxidant effects, demonstrating potential as a feed additive.

Meanwhile, according to “China's Catalogue of Feed Additives (2013)”, food flavorings are permitted to be used as feed seasonings and attractants. As a traditional local spice, *E. cypriani* has a foundation for compliance application. Its resource utilization not only aids antibiotic substitution but may also bring significant economic benefits. Based on the fact that *E. cypriani* is rich in active components such as citral and flavonoids, and has been demonstrated by multiple studies to possess significant antioxidant, anti-inflammatory, and broad-spectrum antibacterial activities, this study proposes the following scientific hypothesis: The inclusion of wild herb powder in the diet can effectively regulate the intestinal microenvironment of Muscovy ducks through its overall biological activity, enhance immune and antioxidant functions, and consequently exert positive effects on growth performance and overall health. Therefore, this study aims to investigate the volatile oil composition of *E. cypriani* and the effects of its herb powder as a feed additive on the growth performance, slaughter performance, cecal short-chain fatty acid content, and microbial community structure of medium-sized white-feathered Muscovy ducks. This research seeks to provide scientific evidence for the application of this plant in the healthy farming of poultry.

## Materials and methods

### Reagents

FeSO₄, H₂O₂, and salicylic acid were obtained from Tianjin Damiao Chemical Reagent Factory; Trolox, 3-Ethyl-benzothiazoline-6-sulfonic acid (ABTS), 1,1-Diphenyl-2-nitrosohydrazide (DPPH), normal alkane standards (C8-C30), and dimethyl sulfoxide (DMSO) were obtained from Sigma-Aldrich Chemical Co. (St. Louis, Missouri). Citral, linalool, nerol, geraniol, and β-caryophyllene (Macklin Reagent Co., Shanghai, China).

### Plant and animal materials and diets

*Elsholtzia cypriani* (Pavol.) S. Chow ex P. S. Hsu was sourced from Zhaotong City, Yunnan Province. Professor Pu Chunxia of Yunnan University of Chinese Medicine identified the plant material used in this experiment.

The experimental diets consisted of a basal diet and a basal diet supplemented with 4 % *E. cypriani* fragrance powder. The supplemented basal diet was prepared by drying and grinding the fresh aerial parts of *E. cypriani* fragrance, processed by Fujian Hualong Feed Co., Ltd. in Zhangzhou City. The experimental animals were medium-sized white-feathered Muscovy ducks supplied by Fujian Changlong Agriculture and Animal Husbandry Co., Ltd. in Zhangzhou City.

### Extraction of essential oils

Following the distillation apparatus specified in the Chinese Pharmacopoeia, volatile oils were extracted via water distillation. For the methods of essential oil extraction, GC-MS detection conditions, and protocols, please refer to the **Supplementary information**.

### Detection of antioxidant activity of E. cypriani essential oil and its major constituents

The in vitro antioxidant activity of *E. cypriani* essential oil was evaluated through DPPH, ABTS, and hydroxyl radical scavenging activity assays. Detailed experimental procedures can be found in the **Supplementary information**.

### Bacteriostatic activity assay

This bacteriostatic assay employed the microdilution method to determine the OD values of bacterial suspensions, evaluating the inhibitory activity of the sample against *Escherichia coli* and *Salmonella anatum*. Detailed experimental procedures can be found in the **Supplementary information**.

Inhibition Rate (%) = [1 - (OD_(experimental) - average OD_(blank)) / (OD_(negative control) - average OD_(blank))) × 100 %

### Animal experimental design and feed formulation

252 21-day-old medium-sized white-feathered Muscovy ducks, all females, were randomly assigned to a basal diet control group (CON) and a group supplemented with 4 % *E. cypriani* grass powder (ECP). Each group had six replicates, with 21 ducks per replicate. Diet composition and nutritional levels are detailed in Table 1. On day 41, three Muscovy ducks were selected from each replicate, electro-stunned, and euthanized by venous bleeding. Slaughter performance and immune organ indices were then measured. All experimental animals were reared in floor-based housing systems under standard feeding management. After sample collection, the carcasses of the Muscovy ducks were subjected to harmless disposal. The experimental protocol, methodologies, observational indicators, and euthanasia procedures complied with ethical requirements for animal experimentation.

**Table 1:** Composition and nutrient levels of experimental diets.

Items	Diet content (%)	
	CON	ECP
Ingredients		
Corn	43.4	38.1
Wheat	25.0	25.0
Double-low rapeseed meal	14.8	12.0
Corn sugar residue A	2.5	2.5
Corn starch residue	8.0	8.0
Wheat middlings	0.157	0.157
Corn protein powder	1.5	1.5
Fine limestone powder	1.4	1.4
Expanded soybean 1	—	3.3
*E. cypriani*	—	4.0
Chicken fat^1^	0.7	1.5
Others^2^	—	—
Total	100	100
Nutritional Level		
ME (MJ/kg)	2.85	2.85
Crude Protein	16.02	16.01
Crude Fiber	4.6	5.62
Crude Fat	4.14	5.36
Crude Ash	5.14	5.32
Electrolyte Balance dEB (mmol/kg)	155.6	165.5
Linoleic Acid	1.78	2.1
Total Phosphorus	0.57	0.55
Calcium	0.84	0.85
Lysine	0.95	0.95

Note:.

1 Energy levels in the CON and ECP groups were balanced by adding expanded soybeans and chicken fat;.

2 Other additives included calcium hydrogen phosphate, sodium chloride (table salt), l-lysine sulfate, DL-methionine, l-threonine, 60 % choline chloride, poultry multivitamins, organic poultry minerals, compound enzyme 808B, heat-resistant phytase, Tianli Golden Yellow, 2.5 % Tianli Red, Baiweisu, and Weishengbao. The addition levels of each component were consistent across both groups.

### Experimental measurement indicators and methods

#### Growth performance measurement

In the experiment, body weight (BW) was measured for each replicate group of Muscovy ducks at 21 and 41 days of age. Based on these measurements, average daily gain (ADG), average daily feed intake (ADFI), average feed consumption per bird, feed conversion ratio (FCR), and overall feed-to-weight ratio were calculated. Serum samples were isolated from blood samples collected from six ducks per group and stored at −20°C for subsequent use. After slaughter, carcass traits were measured according to the "Terminology and Measurement Methods for Poultry Production Performance" (NY/T 823-2020), and corresponding slaughter performance indicators were calculated. Immune organs were excised, trimmed of surrounding fat, weighed fresh, and the immune organ index was calculated.

#### Determination of Serum antioxidant and anti-inflammatory indicators in muscovy ducks

At 41 days of age, venous blood was collected from the Muscovy ducks and placed in sterile tubes. The levels of superoxide dismutase (SOD), malondialdehyde (MDA), glutathione peroxidase (GSH-Px), and catalase (CAT) in the serum were measured according to the instructions of the reagent kits provided by Beijing Leagene Biotechnology Co., Ltd. Additionally, the levels of interleukin-6 (IL-6), tumor necrosis factor-α (TNF-α), and interleukin-1β (IL-1β) in the serum were determined following the instructions of the reagent kits from Shanghai Fenxi Biotechnology Co., Ltd.

#### Cecal microbiome and short-chain fatty acid level assay

Six experimental ducks were randomly selected from each treatment group. After euthanasia via corona bleeding, approximately 0.5 g of cecal contents were collected into 2-mL sterile cryogenic tubes. Samples were preserved with dry ice and shipped to Shanghai Paisenuo Biotechnology Co., Ltd. for analysis of cecal microbial diversity and short-chain fatty acid levels. PCR amplification of the V3-V4 hypervariable region was performed using universal 16S rRNA primers (338F: 5′-ACTCCTACGGGAGGCAGCAG-3′ and 806R: 5′-GGA CTACHVGGGTWTCTAAT-3′) followed by microbial community analysis. Using GC-MS coupled technology, standard curves were established to quantitatively determine the concentrations of valeric acid, isovaleric acid, caproic acid, propionic acid, and butyric acid in cecal contents.

### Statistical analysis

Univariate analysis of variance (ANOVA) was performed using SPSS 15.0 software for growth performance, slaughter performance, and immune organ indices. Results are presented as mean ± standard error. ASV abundance matrices were obtained, and species were annotated using the DADA2 method for denoising based on second-generation sequencing provided by Paisenuo Company. Cloud-based platforms analyzed gut microbiota species composition, alpha diversity, and beta diversity for the two experimental duck groups. GraphPad Prism 9.0 and R 4.3.3 were used to visualize the results. Based on the quantitative detection results of fatty acid levels in the cecum provided by Paisenno Company, t-tests and graphical representations were performed using GraphPad Prism 9.0 software. Differences were considered significant at *P* < 0.05 and highly significant at *P* < 0.01.

## Results and discussion

### Composition analysis of E. cypriani essential oil

The volatile chemical components in the essential oil were identified using GC-MS analysis, combined with computer-aided search against the NIST 20 standard library and relevant literature. Preliminary quantification of each compound was performed using peak area normalization (as detailed in Table 2). A total of 43 volatile components were identified, accounting for 98.65 % of the total chromatographic effluent. The chemical composition is overwhelmingly dominated by monoterpene aldehydes, with geranial (45.09 %) and neral (36.29 %) as the two core constituents. Their combined content reaches 81.38 %, forming the characteristic intense lemon-like scent base of this essential oil. Other key components with contents greater than 1 % include caryophyllene (2.91 %), nerol (2.27 %), isocitral (1.61 %), linalool (1.27 %), isoneral (1.12 %), and humulene (1.01 %). Additionally, dozens of other compounds—terpenes, ketones, and oxides—were detected at lower levels (most below 1 %), collectively contributing to the essential oil's complex and diverse chemical profile. These components have been demonstrated to possess significant biological activity, such as linalool and geranial exhibiting broad-spectrum antibacterial effects (Gao et al., 2019; Gutiérrez-Pacheco et al., 2023), while geraniol and nerol display strong antioxidant properties (Rajendran et al., 2024; Wang et al., 2019).

**Table 2:** Volatile compounds and relative percentages of volatile oils from *E. cypriani.*

No.	Compounds	CAS	RI (Cal)	RI (List)	Match	Relative Content (%)
1	Benzaldehyde	100-52-7	966	962	964	0.06
2	pinene	3387-41-5	977	974	962	0.17
3	sulcatone	110-93-0	986	986	921	0.21
4	β-Myrcene	123-35-3	992	991	852	0.06
5	alpha-Terpinene	99-86-5	1020	1017	909	0.02
6	p-Cymene	99-87-6	1028	1025	846	0.01
7	β-Phellandrene	555-10-2	1033	1031	922	0.06
8	5-Heptenal, 2,6-dimethyl-	106-72-9	1055	1054	915	0.03
9	Linalool oxide	5989-33-3	1076	1074	944	0.09
10	Sabinene hydrate	546-79-2	1070	1075	802	0.27
11	trans-Linalool oxide (furanoid)	34995-77-2	1092	1086	946	0.07
12	Linalool	78-70-6	1101	1099	960	1.27
13	cis-4-(Isopropyl)-1-Methylcyclohex-2-En-1-Ol	29803-82-5	1126	1122	899	0.08
14	7-methyl-3-methyleneoct-6-enal	55050-40-3	1146	1147	923	0.20
15	chrysanthemyl alcohol	18383-59-0	1154	1160	814	0.49
16	Isoneral	72203-97-5	1165	1170	960	1.12
17	rose furan epoxide	92356-06-4	1177	1176	912	0.06
18	Isocitral	55722-59-3	1183	1184	933	1.61
19	α-Terpineol	98-55-5	1196	1189	952	0.22
20	(E)-piperitol	16721-39-4	1212	1208	876	0.16
21	(-)-trans-Isopiperitenol	74410-00-7	1207	1210	878	0.09
22	Nerol	106-25-2	1231	1228	949	2.27
23	Neral	106-26-3	1246	1240	961	36.29
24	Geraniol	106-24-1	1256	1255	975	0.95
25	Geranial	141-27-5	1276	1270	963	45.09
26	Phenol, 2-methyl-5-(1-methylethyl)-	499-75-2	1294	1299	909	0.2
27	2H-1-Benzopyran, 3,4,4a,5,6,8a-hexahydro-2,5,5,8a-tetramethyl-, (2α,4aα,8aα)-	41678-32-4	1299	1318	882	0.12
28	Geranic acid	459-80-3	1352	1355	800	0.08
29	Eugenol	97-53-0	1364	1358	859	0.06
30	Copaene	3856-25-5	1387	1376	896	0.08
31	(-)-β-Bourbonene	5208-59-3	1396	1384	824	0.04
32	Caryophyllene	87-44-5	1433	1419	971	2.91
33	2,6,10-Trimethyltridecane	3891-99-4	1462	1449	925	0.06
34	Humulene	6753-98-6	1467	1454	948	1.01
35	Germacrene D	23986-74-5	1494	1481	953	0.76
36	Spathulenol	6750-60-3	1592	1576	874	0.09
37	Caryophyllene oxide	1139-30-6	1596	1581	904	0.17
38	Humulene epoxide I	19888-33-6	1616	1604	933	0.27
39	humulene epoxide ii	19888-34-7	1626	1606	940	0.21
40	11,11-Dimethyl-4,8-dimethylenebicyclo[7.2.0]undecan-3-ol	79580-01-1	1653	1646	948	0.44
41	Humulenol-II	19888-00-7	1649	1650	828	0.29
42	14-Hydroxycaryophyllene	50277-33-3	1673	1686	880	0.45
43	2-Pentadecanone, 6,10,14-trimethyl-	502-69-2	1848	1844	948	0.48

### Detection of antioxidant activity of e. cypriani essential oil and its major constituents

**Fig. 1:**
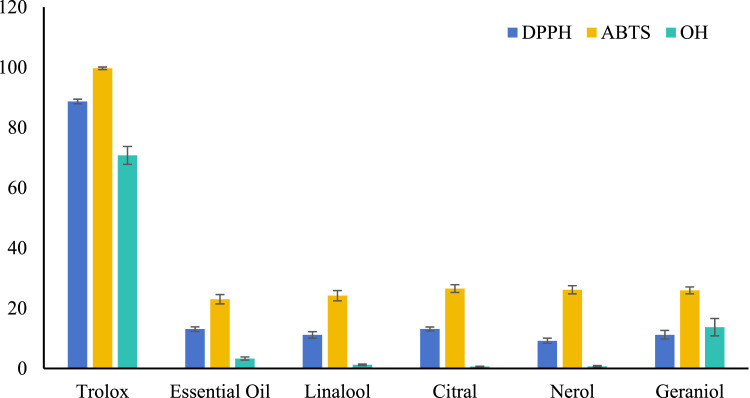
Radical scavenging rate of volatile oils and principal component from *E. cypriani*.

To investigate the antioxidant potential of active components in the *E. cypriani* plant meal, this study first evaluated the free radical scavenging capacity of the volatile oil and major monomeric components through in vitro experiments. The DPPH, ABTS, and hydroxyl radical (OH⁻) scavenging assays are classical methods for assessing direct antioxidant activity. As shown in Fig. 1, at a concentration of 100 μg/mL, the volatile oil of *E. cypriani* and its monomeric components (linalool, citral, nerol, geraniol) generally exhibited weak free radical scavenging capacity. The scavenging rates of ABTS (22.98 %–26.54 %) were relatively higher than those of DPPH (9.21 %–13.11 %), while the hydroxyl radical scavenging capacity was the lowest (0.67 %–13.72 %). Notably, although its direct free radical scavenging activity is not high, geraniol demonstrated the relatively best activity (13.72 %) in the hydroxyl radical scavenging assay, suggesting that its biological activity may be achieved by regulating the endogenous defense system (Srivastava et al., 2024).

The in vitro activity results indicate that the active components in *E. cypriani* may not primarily exert their effects through direct free radical scavenging mechanisms. This finding prompted further investigation into whether they enhance the body's antioxidant status in vivo via other pathways. Therefore, we subsequently conducted a feeding experiment with Muscovy ducks, focusing on the analysis of serum antioxidant enzyme activities and oxidative damage indicators, to clarify the actual antioxidant effects and potential mechanisms of action of this plant meal in animals. This comprehensive evaluation contributes to assessing the antioxidant potential of *E. cypriani* meal as a feed additive.

### Antibacterial activity of E. cypriani essential oil

The primary antibacterial active compounds in aromatic plants are aromatic secondary metabolites, such as plant essential oils. The antibacterial activity of *E. cypriani* essential oil against *E. coli* and *S. enterica* was determined. At a concentration of 6080 μg/mL, *E. cypriani* essential oil exhibited significant inhibitory activity against both *E. coli* and *S. enterica*. Their MIC values were 380 μg/mL and 3040 μg/mL, respectively (as detailed in Table 3). This selective antibacterial activity may be related to differences in bacterial cell wall structure, particularly the permeability of the outer membrane in Gram-negative bacteria(Angane, Swift, Huang, Butts, and Quek, 2022).

**Table 3:** Antibacterial activity of essential oils from *E. cypriani.*

Sample	Concentration (μg/mL)	Antibacterial rate (%)		MIC (μg/mL)	
		*E. coli*	*S. anatum*	*E. coli*	*S. anatum*
Gentamicin^1^	5	101.62 ± 0.12	102.35 ± 0.80	—	—
Essential oil of *E. cypriani*	6080	103.06 ± 3.65	103.55 ± 1.35	380	3040

1 Gentamicin is a positive control.

### Effects of adding E. cypriani on growth performance of muscovy ducks

Twenty-one-day-old medium-sized white-feathered Muscovy ducks were used as experimental subjects. A blank control group (CON group) and an experimental group (ECP group) were established, supplemented with 4 % *E. cypriani* grass powder in the diet. The 40-day trial period measured growth performance, slaughter performance, immune organ indices, cecal microbiota structure, and short-chain fatty acid levels in both groups. Comparative analysis of these metrics evaluated *E. cypriani*'s potential as a plant-based feed additive. As shown in Table 4, the evaluation results indicated that the addition of 4 % *E. cypriani* grass powder exhibited a specific palatability-enhancing effect. After 40 days of feeding, the ECP group demonstrated higher average daily gain, total weight gain, final body weight, total feed intake, and average feed consumption per duck compared to the CON group. However, no significant differences were observed between the two groups in feed conversion ratio or total feed-to-weight ratio (*P* > 0.05). The supplementation had no significant effect on the growth performance of Muscovy ducks (*P* > 0.05).

**Table 4:** The effect of 4 % added *E. cypriani* on the growth performance of experimental ducks.

Items	CON	ECP	F	P
Final body weight (g)	1917.17 ± 20.46	1973.17 ± 18.72	4.077	0.071
Weight gain (g)	1575.17 ± 20.48	1631.00 ± 18.82	4.029	0.073
Total feed intake (kg)	4.18 ± 0.053	4.31 ± 0.031	4.262	0.066
Average daily gain (g)	104.48 ± 1.31	107.63 ± 0.78	4.286	0.065
Average feed conversion ratio (g)	202.22 ± 2.54	208.33 ± 1.50	4.281	0.065
Feed conversion ratio	2.65 ± 0.02	2.64 ± 0.02	0.270	0.614
Feed-to-weight ratio	2.18 ± 0.02	2.18 ± 0.01	0.008	0.928
Final quantity	124	124	—	—

### Effects of adding E. cypriani on slaughter performance of muscovy ducks

Slaughter performance is a key method for evaluating carcass quality (Li et al., 2021). After 40 days of feeding, two experimental ducks (with weights close to the group average) were selected from each replicate, totaling 12 ducks per group, for assessment of slaughter performance. Results indicated that adding 4 % *E. cypriani* grass powder to the diet increased the pre-slaughter live weight, slaughter weight, and half-gutted weight (with heart, liver, and kidneys retained), slaughter yield, abdominal fat percentage, and skin fat percentage. However, compared to the CON group, the ECP group exhibited lower total dressed weight (*P* > 0.05) and total dressed yield (*P* > 0.05), as shown in Table 5. The differences between CON and ECP groups for live weight and slaughter weight were 250.50 ± 3.27 g and 239.08 ± 8.03 g, respectively; while for slaughter weight and half-gutted weight were 216.75 ± 7.28 g and 228.17 ± 8.72 g, respectively. The differences between the CON and ECP groups for the semi-gutted weight and the fully gutted weight were 85.08 ± 3.53 g and 110.75 ± 6.52 g, respectively. Through one-way ANOVA, adding 4 % *E. cypriani* grass powder had a highly significant effect (*P* < 0.01) on the difference between the semi-gutted weight and the fully gutted weight of the experimental Muscovy ducks. The antibacterial activity of essential oils reduces the burden of intestinal pathogens, improving the digestion and absorption of nutrients(Abd El-Hack et al., 2022). Furthermore, their antioxidant and anti-inflammatory effects lower metabolic stress in the body, making more nutrients available for muscle growth (He et al., 2023; Johnson et al., 2025). Additionally, we observed a moderate increase in muscle fat content, which may be related to the essential oil components' regulation of lipid metabolism (Popović et al., 2016). This suggests that the supplementation of 4 % *E. cypriani* grass powder may influence the development of the heart, liver, glandular stomach, and lungs, as well as abdominal fat formation in Muscovy ducks.

**Table 5:** The effect of 4 % added *E. cypriani* on the slaughtering performance of experimental ducks.

Items	CON	ECP	F	P
Half-carcass weight	1432.67 ± 10.39	1445.08 ± 9.63	0.768	0.390
Whole-carcass weight	1347.58 ± 9.84	1334.33 ± 10.13	0.880	0.358
Belly fat weight	50.25 ± 2.57	57.00 ± 3.92	2.071	0.164
Skin fat weight	277.33 ± 4.47	284.25 ± 6.46	0.774	0.389
Slaughter yield	86.81 ± 0.17	87.50 ± 0.41	2.283	0.145
Half-carcass yield	75.41 ± 0.44	75.58 ± 0.55	0.058	0.812
Whole-carcass yield	70.93 ± 0.48	69.78 ± 0.56	2.418	0.133
Breast meat yield	13.83 ± 0.24	13.72 ± 0.32	0.074	0.788
Leg meat yield	16.86 ± 0.44	16.06 ± 0.43	1.671	0.210
Belly fat yield	3.60 ± 0.19	4.10 ± 0.28	2.197	0.152
Skin fat yield	23.44 ± 0.44	24.54 ± 0.62	2.076	0.164
(Live weight - Slaughter weight)	250.50 ± 3.27	239.08 ± 8.03	1.732	0.202
(Slaughter weight - Half-carcass weight)	216.75 ± 7.28	228.17 ± 8.72	1.011	0.326
(Half-carcass weight - Whole-carcass weight)	85.08 ± 3.53	110.75 ± 6.52	11.969	0.002^⁎⁎^

Note: Live weight, carcass weight, and total dressed weight—unit: grams (g); other units are %. * indicates *P* < 0.05; ** indicates *P* < 0.01.

### Effects of adding E. cypriani on immune organ index in muscovy ducks

The immune organ index is a non-specific immune parameter. The development of immune function is closely linked to growth performance, morbidity, and mortality, and the immune organ index can always be used to reflect the level of immune response in poultry (Zhu et al., 2023). After 40 days of feeding, one Muscovy duck (with a weight close to the average) was randomly selected from each replicate group. The ducks were euthanized by electrocution followed by exsanguination. The cecum contents were collected, and the bursa of Fabricius, spleen, and thymus were excised and weighed from the experimental ducks to determine the immune organ indices of the Muscovy ducks. The results are shown in Table 6. Compared with the CON group, the ECP group exhibited increased cecal and thymus index. Single-factor ANOVA revealed a highly significant difference (*P* < 0.01) in thymus indices between the CON and ECP groups. The main function of the thymus is to produce T lymphocytes and secrete thymosin, playing a key role in cellular immunity (Thapa and Farber, 2019). Supplementing with 4 % *E. cypriani* powder exerted a highly significant effect (*P* < 0.01) on the thymus index of experimental Muscovy ducks, suggesting the herb powder significantly promotes the development of immune organs. Plant essential oils can enhance immune organ development by regulating the neuroendocrine-immune network (Wang et al., 2024). Mechanistically, terpenoid components in essential oils may influence the hypothalamic-pituitary-adrenal axis to regulate immune cell proliferation and differentiation (Siddiqui et al., 2024). The maturation of immune organs provides a structural foundation for disease resistance, which is a key reason for the overall health improvement observed in the experimental group of Muscovy ducks (Kogut, 2009).

**Table 6:** The effect of 4 % added *E. cypriani* on the immune organ index of experimental ducks.

Items	CON	ECP	F	P
Follicular Index (%)	1.69 ± 0.14	2.03 ± 0.32	0.888	0.368
Spleen Index (%)	1.38 ± 0.10	1.31 ± 0.08	0.280	0.608
Thymus Index (%)	4.55 ± 0.24	6.04 ± 0.33	13.479	0.004**

Note: * indicates *P* < 0.05; ** indicates *P* < 0.01.

### Effects of Adding E. cypriani on serum antioxidant and anti-inflammatory indicators in muscovy ducks

The determination of antioxidant and anti-inflammatory indicators is crucial for assessing animal health status and the efficacy of nutritional interventions. Oxidative stress refers to an imbalance between oxidation and antioxidation in the body, which can generate various reactive oxygen species (ROS) that damage proteins, nucleic acids, and lipids. It is closely associated with reduced growth performance, weakened immunity, and deteriorated product quality in animals (Celi, 2010). Malondialdehyde (MDA), as a terminal product of lipid peroxidation, directly reflects the extent of cellular membrane damage caused by free radical attacks (Durand et al., 2022). In Table 7, the MDA content in the experimental group was significantly lower than that in the control group (*p* < 0.001), indicating that the addition of *E. cypriani* meal effectively reduced the level of lipid peroxidation in the body.

**Table 7:** Antioxidant and anti-inflammatory indicators in muscovy duck serum.

Items	CON	ECP	P
SOD (U/mL)	64.15 ± 1.58	67.90 ± 2.36	0.216
MDA(μmol/L)	4.86 ± 0.13	3.29 ± 0.25	<0.001⁎⁎⁎
GSH-Px (U/mL)	557.33 ± 12.27	632.00 ± 11.31	0.001⁎⁎
CAT (U/mL)	2.93 ± 0.11	2.69 ± 0.09	0.122
IL-1β(pg/mL)	14.41 ± 0.56	12.95 ± 0.24	0.036*
TNF-α(pg/mL)	7.13 ± 0.48	5.57 ± 0.72	0.043*
IL-6(pg/mL)	34.11 ± 2.14	26.32 ± 2.06	0.026*

Note:.

⁎ indicates *P* < 0.05;.

⁎⁎ indicates *P* < 0.01;.

⁎⁎⁎ indicates *P* < 0.001.

Glutathione peroxidase (GSH-Px) is a key enzyme responsible for scavenging hydrogen peroxide and lipid peroxides (Durand et al., 2022). The activity of GSH-Px was significantly elevated in the experimental group (*p* = 0.001), suggesting that the herb meal enhanced this critical antioxidant defense mechanism. In contrast, no significant differences were observed in the activities of superoxide dismutase (SOD) and catalase (CAT) between the control and experimental groups (*p* > 0.05), indicating that *E. cypriani* meal may exert its antioxidant effects through specific modulation of the GSH-Px pathway. This finding is corroborated by other studies in poultry nutrition. For instance, research on broilers under heat stress conditions revealed that supplementing with exogenous antioxidants effectively suppressed oxidation and protected meat quality (Vakili and Rashidi, 2011). This fully demonstrates the critical importance of strengthening the body's antioxidant defense system. Vakili et al. (2010) found that antioxidants in feed significantly improved weight gain and feed conversion ratio in female broilers. In summary, the enhanced weight gain observed in Muscovy ducks in this study likely stems from the antioxidant activity of essential oil components in wild herb powder, as evidenced by improved serum antioxidant markers. This finding aligns closely with the established logic chain of “antioxidant activity improving production performance”revealed in the aforementioned research.

Inflammation is a core pathological process in response to injury, while chronic inflammation can lead to nutrient redistribution and inhibit growth and development. Tumor necrosis factor-α (TNF-α) and interleukin-1β (IL-1β) are initiators of inflammatory responses and can induce inflammatory cascades (Docherty et al., 2022; Thoma and Lightfoot, 2018). The levels of these two cytokines were significantly reduced in the experimental group (*p* < 0.05), demonstrating that the herb meal suppressed the activation of inflammatory responses from the source. Interleukin-6 (IL-6), as an essential pro-inflammatory cytokine, participates in acute-phase reactions and promotes inflammation progression (Basiouni et al., 2023). The significantly decreased IL-6 level in the experimental group (*p* < 0.05) further confirmed the anti-inflammatory effect of the herb meal.

The inclusion of 4 % *E. cypriani* meal in the diet effectively alleviated oxidative damage by specifically enhancing the GSH-Px-mediated antioxidant pathway and further inhibited the release of pro-inflammatory cytokines, thereby comprehensively improving the antioxidant and anti-inflammatory capacities of Muscovy ducks. The synergistic effects of these antioxidant and anti-inflammatory actions provide a theoretical basis for using *E. cypriani* meal as a functional feed additive to enhance animal health and production performance. These findings suggest that *E. cypriani* herb powder may upregulate endogenous antioxidant enzyme expression by activating the Nrf2-ARE signaling pathway and alleviate inflammatory responses by inhibiting the NF-κB pathway. Notably, this endogenous activation mechanism contrasts sharply with its relatively weak direct radical scavenging capacity in vitro, suggesting its mode of action focuses more on regulating the body's defense systems rather than directly neutralizing free radicals.

### Effects of adding E. cypriani on short-chain fatty acid levels in the cecum of muscovy ducks

**Fig. 2:**
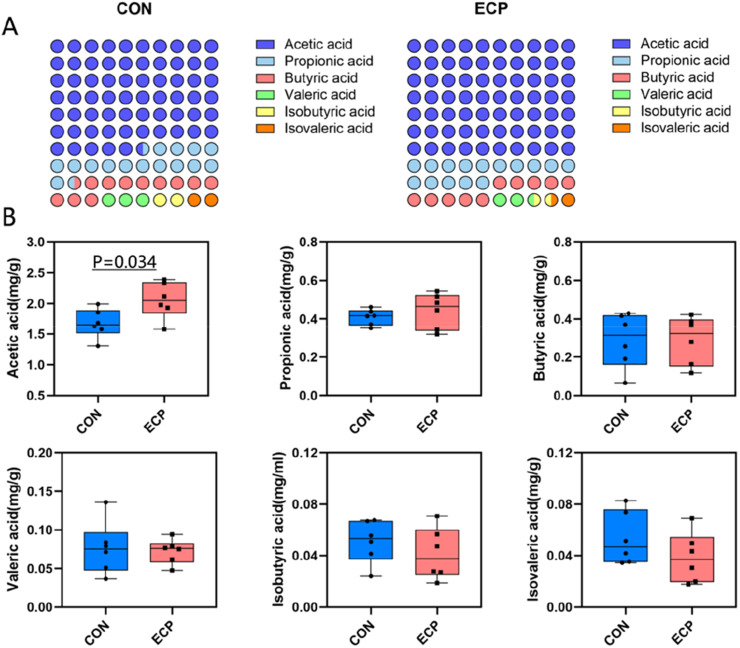
Profile of short-chain fatty acids (SCFAs) in the cecum of the CON and ECP groups (A) and intergroup t-test (B).

Host intestinal homeostasis and health depend on the gut microbiota. Short-chain fatty acids (SCFAs), produced by gut bacteria during fermentation of undigested fiber and resistant starch, play a crucial regulatory role in maintaining intestinal health (Cong et al., 2022). Growing evidence suggests that the gut microbiota plays a pivotal role in maintaining host health and intestinal homeostasis. This is achieved through the release of SCFAs, the primary bacterial metabolites produced by specific colonic anaerobes fermenting dietary fiber and resistant starch, primarily comprising acetate, propionate, and butyrate. SCFAs formation results from complex interactions between diet and gut microbiota within the intestinal lumen (Portincasa et al., 2022). SCFAs serve as signaling molecules mediating interactions among diet, microbiota, and host, playing vital roles in immune, metabolic, and endocrine functions (Mann et al., 2024). SCFA levels also constitute a key evaluation metric in research on plant-derived feed additives. Therefore, after 40 days of feeding, one Muscovy duck per replicate (with body weight close to the group average) was selected to collect cecal contents. Short-chain fatty acid levels were measured in the CON and ECP groups, and microbial community diversity and relative abundance in the cecal contents of both groups were analyzed via 16S rRNA sequencing. Fig. 2 illustrates the overview of cecal short-chain fatty acid levels in the CON and ECP groups. Compared with the CON group, the ECP group showed a significant trend toward increased cecal acetate concentration (Fig. 2B, *P* < 0.05). Supplementation with 4 % *E. cypriani* grass powder significantly influenced the cecal SCFA composition in experimental Muscovy ducks (*P* < 0.05). This finding is significant because short-chain fatty acids not only provide energy to intestinal epithelial cells but also regulate systemic inflammatory responses. Increased levels of beneficial bacteria such as Lactobacillus promote the production of short-chain fatty acids, which can influence liver metabolism through the "gut-liver axis" and thereby modulate systemic inflammatory states (Smolinska et al., 2025). Furthermore, short-chain fatty acids enhance intestinal barrier function and reduce endotoxin entry into the bloodstream, which may be a key reason for the improvement in systemic inflammatory markers.

### Effects of adding E. cypriani on microbial structure and community in the cecum of muscovy ducks

**Fig. 3:**
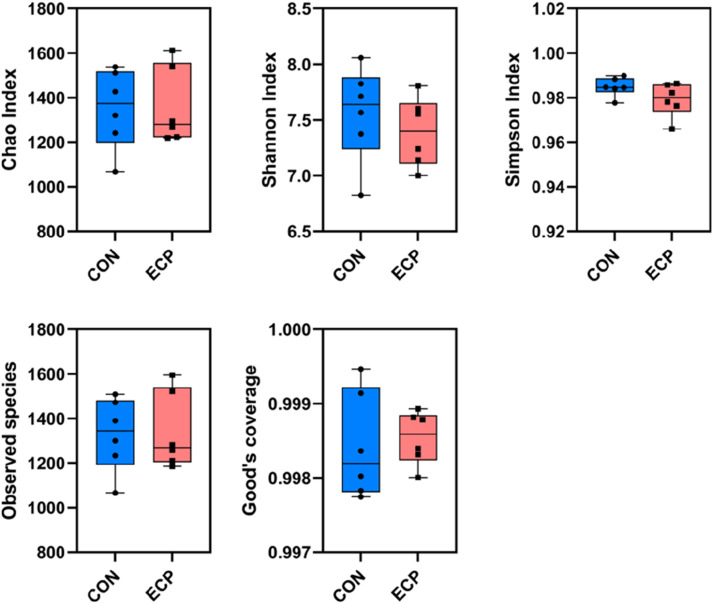
Inter-group t-test of the α-diversity index.

**Fig. 4:**
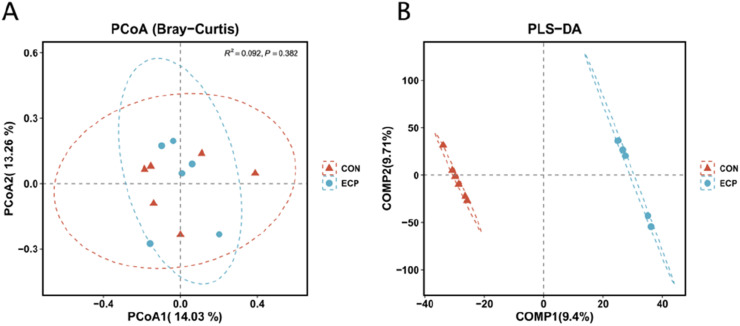
Principal coordinates analysis (A) and partial least squares-discriminant analysis (B).

A vast number of microorganisms inhabit the poultry gut, regulating growth and development, immunity, and stress resistance of the body (Aruwa, Pillay, Nyaga, and Sabiu, 2021). As ducks grow and develop, gut microbial diversity increases and stability enhances, reflecting the gradual stabilization and maturation of the gut microbiota during host maturation (Zhang et al., 2023). The study indicates that microencapsulating multiple plant essential oils (such as thyme and peppermint) effectively preserves their bioactivity and significantly improves broiler chickens' growth performance, antioxidant status, and gut health. This provides a reliable reference for the application of plant essential oils in animal husbandry (Moharreri et al., 2022). Alpha diversity analysis of caecal microbiota between the two groups was conducted using the Chao 1 index, Observed species index, Shannon index, Simpson index, and Good's coverage index to assess caecal microbial richness, diversity, and character coverage in the CON and ECP groups. Comparison via t-tests revealed no significant differences in alpha diversity indices between groups (*P* > 0.05), as shown in Fig. 3. Beta diversity indices focus on comparing species diversity between different habitats, i.e., differences between samples. As shown in Fig. 4A, adding 4 % *E. cypriani* grass powder had no significant effect on caecal microbial diversity compared to the CON group. However, partial least squares regression (Fig. 4B) revealed differences in caecal microbial community structure between the CON and ECP groups.

**Fig. 5:**
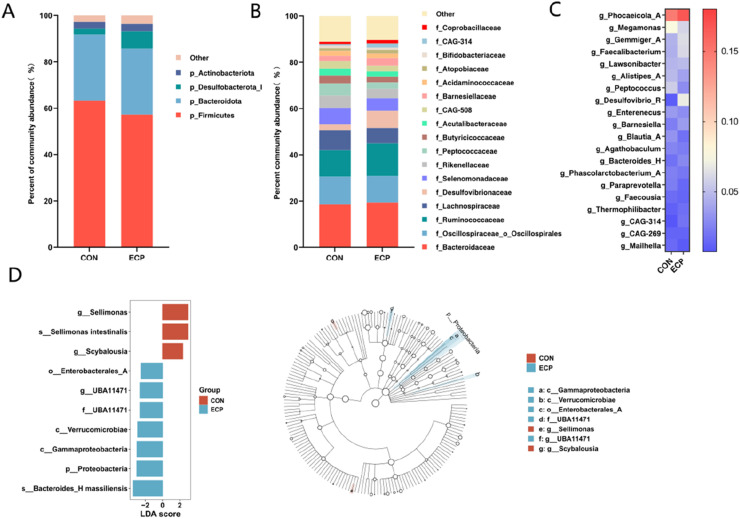
Composition at the phylum (A), family (B), and genus (C) levels and LEfSe analyses (D).

In the cecal microbial communities of experimental Muscovy ducks from both groups, the phyla Bacteroidetes and Firmicutes dominated, accounting for approximately 90 % of the total composition (Fig. 5A). At the family level, the dominant genera were *Bacteroidaceae, Oscillospiraceae_o_Oscillospiraes, Ruminococcaceae, Lachnospiraceae, Desulfovibrionaceae, Selenomonadacea*e, *Rikenellacea*e, and *Peptococcaceae* (Fig. 5B). At the genus level, dominant genera included *Phocaeicola_A, Megamonas, Gemmiger_A, Faecalibacterium, Lawsonibacter, Alistipes_A, Peptococcus, and Desulfovibrio* (Fig. 5C). LEfSe analysis revealed (Fig. 5D) distinct bacterial communities in the cecum between the two treatments. At the genus level, the CON group showed abundant *Sellimonas* and *Scybalousia*, while the ECP group exhibited abundant UBA11471 (*c_Bacteroidia; o_Bacteroidales; f_UBA11471; g_UBA11471*, belonging to the *Bacteroidetes* phylum; based on genomic sequence prediction, it may possess genes for degrading specific polysaccharides). At the species level, the CON group showed enrichment of *Sellimonas intestinalis* (a key butyrate-producing bacterium in the gut. Butyrate is vital for intestinal health, serving as the primary energy source for colonic epithelial cells and exerting anti-inflammatory effects while maintaining intestinal barrier integrity (McKee, La Rosa, Westereng, Eijsink, Pope, and Larsbrink, 2021). The ECP group showed enrichment of *Bacteroides_H massiliensis,* renowned for potent polysaccharide degradation capabilities. These bacteria break down complex dietary polysaccharides and oligosaccharides indigestible by the host, fermenting them into short-chain fatty acids like acetate and succinate to provide energy for the host (Yu et al., 2025). *Bacteroides* can degrade complex plant polysaccharides such as starch, cellulose, xylan, and pectin, producing SCFAs like succinate, acetate, and butyrate through carbohydrate metabolic pathways (Fan, Ju, Bhardwaj, Korver Douglas, and Willing Benjamin, 2023; McKee et al., 2021). The presence of differential bacterial species between the ECP and CON groups in the cecum may potentially correlate with elevated cecal acetate levels in the ECP group, warranting further investigation and discussion.

## Conclusions

This study detected the main components of *E. cypriani* essential oil via GC-MS as geranial (45.09 %) and neral (36.29 %). Their known antibacterial, anti-inflammatory, and antioxidant properties provide the material basis for subsequent holistic effects. Although adding 4 % of this powder to feed did not significantly increase duck growth rate or meat yield, it successfully stimulated appetite. Its most pronounced effect manifests in comprehensive health improvement. By elevating serum antioxidant enzyme (GSH-Px) activity while reducing oxidative damage markers (MDA) and pro-inflammatory cytokine levels (IL-1β, TNF-α, IL-6), it significantly enhanced the body's antioxidant and anti-inflammatory capabilities. Concurrently, the thymus index of treated ducks markedly increased, indicating enhanced development of immune organs. This systemic health improvement was closely linked to gut regulation, as the additive reshaped the intestinal microbiota by increasing the abundance of fiber-degrading bacteria and elevating acetate levels. This created a more favorable microenvironment for immune system development and functional maintenance. In summary, *E. cypriani*, rich in geraniol and nerolidol, effectively supports overall duck health primarily through its active components' antioxidant properties, gut microbiota regulation, and anti-inflammatory effects. These findings lay a theoretical foundation for developing green feed additives as potential alternatives to antibiotics.

## CRediT authorship contribution statement

**HuiWei Zhou:** Writing – original draft, Visualization, Data curation. **XinYe Tian:** Writing – original draft, Visualization, Formal analysis, Data curation. **YongPeng Ma:** Resources, Investigation. **AZheng Liang:** Resources, Funding acquisition, Formal analysis. **XiaoDong Zhuang:** Resources, Methodology, Funding acquisition. **ZhiZhi Du:** Writing – review & editing, Supervision, Methodology, Funding acquisition.

## Disclosures

The authors declare that they have no known competing financial interests or personal relationships that could have appeared to influence the work reported in this paper.

## Appendix Supplementary materials

Image, application 1
