# Supplementary material for: Dietary supplementation with herbal powder of Elsholtzia cypriani improves immune function in Muscovy ducks by activating endogenous antioxidant system and modulating gut microbiota
Source: Poult Sci. 2025 Nov 8;104(12):106080. doi: 10.1016/j.psj.2025.106080 (PMC12663620; doi:10.1016/j.psj.2025.106080)
Supplement: Supplementary file 1 [file mmc1.docx]

**Supplementary information**

*Extraction of Essential Oils*

One hundred grams of dried *E. cypriani* plant material was cut into segments and placed in a round-bottom flask. Two liters of water were added, and the mixture underwent water distillation for 4 hours to extract the volatile oils. Anhydrous sodium sulfate was added for drying. After allowing the mixture to dry, the volatile oils were transferred to a glass bottle and stored at 4°C.

*Gas Chromatography-Mass Spectrometry (GC-MS) Analysis and Volatile Compound Identification*

The essential oil was dissolved in dichloromethane to prepare a 0.2% (v/v) solution. Analysis was performed using a gas chromatography-mass spectrometry (GC-MS, Model 7890A-5795C, Agilent Technologies, Palo Alto, CA, USA) with the following injection method. Column: Agilent 19091J-115, HP-5 (5% Phenyl Methyl Siloxane, 50 m × 0.32 mm × 0.52 μm); Injection volume: 1 μL; Inlet temperature: 250 °C; Pressure: 82.134 kPa; Split ratio: 30:1; Carrier gas: High-purity helium, flow rate: 1.5 mL/min; Column temperature program: Hold at 60°C for 0 min, ramp at 3°C/min to 240°C and hold for 0 min, total run time 60 min; Post Run: 300°C for 5 min, solvent delay 5 min. MS Conditions: Quadrupole temperature 150 °C, ion source temperature 230 °C, EI ionization, electron impact energy 70 eV, scan range 50–550 nm; Qualitative and Quantitative Methods: Qualitative analysis was performed by comparing the mass spectra (MS) and retention indices (RI) with those in the NIST 20 standard mass spectral library using computer searching. The relative percentage content of each chemical component in the volatile oil was quantified via the peak area normalization method.

*DPPH Free Radical Scavenging Activity*

The DPPH free radical scavenging activity of the volatile oil from Wild Mint (or specify the botanical name if available, e.g., Elsholtzia cyprianovi) was measured with some modifications according to the reported method (Xie et al., 2021). Briefly, the reaction mixture consisted of a DPPH solution (100 μM, dissolved in ethanol) and the sample solution. The reaction solutions were mixed and incubated in the dark at 37 °C for 1 hour. Subsequently, the absorbance was measured at 517 nm, using Trolox as the positive control. The DPPH free radical scavenging capacity was calculated using the following formula:

DPPH scavenging activity (%) = [1 − A1 / A0] × 100

Where A0 is the absorbance of the blank control at 517 nm, and A1 is the absorbance of the sample.

*ABTS Free Radical Scavenging Activity*

The ABTS radical cation solution was prepared by dissolving ABTS in ultrapure water to a concentration of 7 mM and mixing it with a 2.45 mM potassium persulfate (K_2_S_2_O_8_) solution at a ratio of 2:1. The solution was kept in the dark at room temperature for 12-16 hours before use. Before measurement, the mixture was diluted with ethanol until the absorbance reached a specific value at 734 nm (Chen et al., 2018). For the experimental group, 20 μL of the sample solution was mixed with 180 μL of the ABTS working solution and allowed to stand at room temperature for 5 minutes. For the background control group, ABTS was not added. Trolox was used as the positive control, and the absorbance was measured at 734 nm (Xie et al., 2021). The ABTS radical scavenging rate was calculated as follows:

ABTS scavenging activity (%) = [1 − (AS − A2) / (A0 - A1)] × 100

Where A0 is the absorbance of the blank control group, A1 is the absorbance of the blank background group, AS is the absorbance of the sample mixed with the ABTS working solution, and A2 is the absorbance of the sample without the ABTS working solution.

*Hydroxyl Radical Scavenging Activity*

This activity assay was performed using the method described by Li et al. with some modifications. For the experimental group, the sample solution, salicylic acid (9 mM), FeSO_4_ (9 mM), and H_2_O_2_ (8.8 mM) were added and thoroughly mixed. For the background group, H_2_O_2_ was replaced with an equivalent volume of ultrapure water. After an incubation period of 30 minutes, the absorbance of the mixture was measured at 510 nm, using Trolox as the positive control. The calculation formula for the hydroxyl radical scavenging activity was the same as that for the ABTS assay.

This bacteriostatic assay employed the microdilution method to determine the OD values of bacterial suspensions, evaluating the inhibitory activity of the sample against *Escherichia coli* and *Salmonella anatum*. Pre-mixed MH broth, sample solutions, and bacterial suspensions were combined to prepare a mixture with a final sample concentration of 60–80 μg/mL, a final concentration of 5 μg/mL for the positive drug control (gentamicin solution), and a final bacterial suspension concentration of 5 × 10⁵ CFU/mL. The experiment included sample groups, medium blank controls, bacterial controls, and gentamicin (positive) controls, each with three replicates. The mixtures were placed in a 96-well plate and incubated at 37°C for 24 hours. The OD values at 630 nm were measured using a microplate reader, and the inhibition rate was calculated using the following formula:

Inhibition Rate (%) = [1 - (OD_(experimental) - average OD_(blank)) / (OD_(negative control) - average OD_(blank))) × 100%
